# The relationship between loneliness and internalizing disorders among young adults: the mediating and moderating role of ego-resilience

**DOI:** 10.3389/fpsyt.2024.1466173

**Published:** 2025-01-03

**Authors:** Anita Padmanabhanunni, Tyrone B. Pretorius

**Affiliations:** Department of Psychology, University of the Western Cape, Cape Town, South Africa

**Keywords:** loneliness, internalizing disorders, depression, anxiety, hopelessness, ego-resilience

## Abstract

**Background:**

The relationship between loneliness and internalizing disorders has been well established in psychological research. This study aims to build on existing research by exploring how different components of loneliness—isolation, relational connectedness, and collective connectedness—interact with ego-resilience to influence anxiety, depression, and hopelessness.

**Methods:**

The study participants were young adults (*n* = 337) who completed the University of California-Los Angeles Loneliness Scale, Ego Resilience Scale, Centre for Epidemiological Studies Depression Scale, State-Trait Anxiety Scale, and Beck Hopelessness Scale. A regression-based moderation and mediation analysis was used to investigate the relationships between the components of loneliness, ego-resilience, and internalizing disorders.

**Results:**

The results of correlational analysis demonstrated that the zero-order correlations between the components of loneliness, isolation, relational connectedness, and collective connectedness, on the one hand, and internalizing disorders, on the other hand, were all significant. Mediation analyses found that ego-resilience partly mediated the relationships between relational connectedness and internalizing disorders, the relationships between collective connectedness and internalizing disorders, and the relationships between isolation and internalizing disorders. Further, moderation analyses found that ego-resilience moderated the relationships between collective connectedness and relational connectedness, respectively, and hopelessness.

**Conclusion:**

This study highlights the central role of ego-resilience in mediating the effects of different facets of loneliness on internalizing disorders. Understanding the mediating and moderating role of ego-resilience can inform therapeutic approaches and interventions aimed at reducing the impact of loneliness on mental health.

## 1 Introduction

Meaningful social connections are central to the maintenance of health and well-being. In the absence of meaningful relationships with others, feelings of loneliness can arise (1). Although transient feelings of loneliness are common, chronic or severe loneliness can precipitate adverse mental and physical health outcomes. Loneliness is defined as an unpleasant affective state evoked in response to perceived discrepancies between desired and actual social connections with others (2). A meta-analysis of studies of loneliness in 113 countries confirmed that loneliness impacts a substantial subset of the global population and is most commonly experienced by young and elderly people (3). In a meta-analytic study of loneliness among older adults in high-income countries, Chawla and colleagues (4) found that loneliness was a common but not universal experience in later life and that factors such as gender, disability status, and living circumstances can influence levels of loneliness. Loneliness has also been documented as a significant concern among children, adolescents, and university students (5–7).

The extant literature base on loneliness has demonstrated its association with social skills deficits, low emotional intelligence, reduced problem-solving efficacy and locus of control, and decreased perceptions of social support (6, 8, 9). Studies have also highlighted the impact of loneliness on physical health indicators, including increased cholesterol, high blood pressure, heightened risk of coronary disease, and vulnerability to cognitive impairments such as dementia (3, 10). Further, loneliness has been consistently associated with adverse mental health outcomes such as anxiety, depression, and hopelessness (9, 11). Although anxiety, depression, and hopelessness are highly correlated with one another, they reflect phenomenologically distinctive constructs. Anxiety is rooted in fear and involves feelings of worry and dread and a sense of apprehension, whereas depression is characterized by consistent feelings of sadness, low motivation, sleep disturbances, and low self-worth, as well as a general dissatisfaction or lack of interest in life (12). Anxiety typically precedes depression, and it has been hypothesized that elevated anxiety may impair interpersonal relationships and prevent an individual from reaching out to others for support. Social withdrawal can amplify depressive symptomology and produce a sense of isolation and loneliness (13).

Hopelessness is conceptualized as a psychological state characterized by a negative outlook toward the future, a sense that life’s problems are insurmountable, and a belief that one’s efforts will not lead to a positive outcome (2). According to hopelessness theory, depressive symptoms are more likely to occur when individuals appraise negative life events as arising from internal, stable, and global causes rather than external causes—essentially attributing these events to personal failings. This cognitive style leads individuals to experience a sense of despair and hopelessness about the future, because they believe that their circumstances are unlikely to improve due to their own unchangeable characteristics. This theory emphasizes the role of negative cognitive attributions in fostering an expectation of hopelessness, which, in turn, increases the risk of developing depressive symptoms (14).

The comorbidity of anxiety and depression, along with the frequency of their concurrent manifestation, can be partially attributed to psychometric issues inherent in self-report scales and taxonomic classifications. However, even when controlling for these factors, measures of the two constructs remain highly intercorrelated (15–17). To account for this intercorrelation, Clark and Watson (17) developed the tripartite model, in which they propose that a specific (negative affect) and non-specific (positive affect) factor or dispositional characteristic explains the strong association between these constructs. Negative affect is considered a common factor of both anxiety and depression and accounts for their strong association, whereas low positive affect is considered distinctive to depression. Anxiety and depression are further distinguished by a cluster of relatively distinctive symptoms. Somatic symptoms and physiological hyperarousal are specific to anxiety, whereas anhedonia and the absence of positive affect are specific to depression (17). A range of studies have provided empirical support for the tripartite model (18–20).

Recent studies have demonstrated that protective factors significantly contribute to variations in susceptibility to mental health conditions. This differential vulnerability explains why some people develop mental health problems while others remain resilient under similar stressful conditions. Factors contributing to differential vulnerability include problem-solving ability, capacity for emotional regulation, intellectual functioning, perceived social support, and sense of coherence (21–23). Understanding these differential vulnerabilities is crucial in developing targeted interventions and prevention strategies tailored to individual risk profiles. The current study focuses on ego-resilience as a protective factor in mental health.

Ego-resilience is defined as a personality trait that involves the adaptive or flexible modulation of emotional impulses depending on the demands of the situation. It is a capacity that supports adjustment to shifting environmental conditions (24). Block (24) formulated the construct of ego-resilience and proposed that it functions as an affective-processing system that enables an individual to respond effectively to environmental changes and stressors. Individuals with higher levels of ego-resilience are believed to be better equipped to manage and recover from stress, due to their ability to remain stable yet flexible in the face of adversity. In contrast, those with lower levels of ego-resilience demonstrate more rigid and stereotypical behavior when confronted with unfamiliar situations or life stressors. A significant literature base supports the role of ego-resilience in mental health. Sanecka and colleagues (25) found that Polish adults with higher levels of ego-resilience were better able to locate and mobilize resources when confronted with life stressors. These authors suggest that high levels of ego-resilience promote a more positive perspective toward adversity (e.g., viewing life stressors as challenges to be overcome), and in turn, this perspective could increase the probability of successfully securing resources for coping and promoting well-being. In a study of adolescent school dropouts, Kwon (26) concluded that ego-resilience mediated the relationship between social stigma and depressive symptoms. Adolescents with higher levels of ego-resilience were better able to navigate the social stigma associated with dropping out of school, and this ability was a mitigating factor against depressive symptoms. Busch and colleagues (27) reported that lower levels of ego-resilience were more strongly associated with anxiety than depression in children. To explain this finding, Busch and colleagues (27) hypothesized that anxiety may impair cognitive processing, which, in turn, impacts the activation of ego-resilience capacities. A Korean study found that ego-resilience was significantly related to suicidal ideation among the elderly. Those with higher levels of ego resilience demonstrated greater cognitive flexibility in navigating life’s problems, and this flexibility buffered against suicidal ideation (28).

There are two potential mechanisms through which ego-resilience can interact with loneliness to influence mental health outcomes. The first mechanism entails a moderating role. In mental health research, a moderator is conceptualized as a third variable that affects the direction or the strength of the association between a predictor variable and a dependent variable (29). In the moderating role, ego-resilience acts to mitigate or buffer the adverse effects of life stressors on an individual’s psychological well-being. In effect, individuals with higher ego-resilience can reduce the impact of stress, allowing them to maintain better mental health in the face of challenges. These individuals are also less likely to succumb to the pressures that typically exacerbate psychological distress, which leads them to have a more stable emotional state. The second mechanism involves a mediating function whereby the third variable represents the mechanism through which the independent variable influences the dependent variable. In the meditating role, ego-resilience transforms the experience of the life stressor into an opportunity for psychological growth, which ultimately has a positive influence on overall mental health (29).

This study was undertaken in the South African context. It aims to build on existing research by exploring how different components of loneliness interact with ego-resilience to influence anxiety, depression, and hopelessness. South African society is characterized by high levels of social fragmentation and inequality. This is ascribed to the legacy of apartheid era policies including forced relocation, which corroded social networks and community ties. In addition, restrictions on the co-residence of family members in urban settings for people of color disrupted family structures and weakened familial bonds (30). Currently, social cohesion in the country is undermined by high levels of poverty, unemployment, violent crime and exposure to traumatic events (31). This may lead to heightened feelings of alienation and loneliness (32) and underscores the importance of examining loneliness and its interaction with key psychological resources such as ego-resilience to better understand their impact on mental health.

The most commonly used instrument for the measurement of loneliness is the University of California Loneliness Scale [UCLA-LS: (33)]. The scale’s dimensionality has been assessed in a range of studies, and the model that has received consistent support consists of three factors—isolation, collective connectedness, and relational connectedness (34, 35). Isolation refers to the subjective experience of feeling alone and disconnected from others. This dimension captures feelings of loneliness and a perceived lack of social relationships or support. Collective connectedness pertains to the sense of belonging and being part of a larger community or social network. It involves the perception of having a support system, shared interests, and meaningful relationships that contribute to one’s social integration. Relational connectedness focuses on the quality and depth of close interpersonal relationships. It encompasses feelings of intimacy, emotional closeness, and strong bonds with significant others, such as family members, friends, and romantic partners (34–36).

While the relationship between loneliness and mental health outcomes is well-documented, few studies have examined how the distinct components of loneliness interact with ego-resilience to influence anxiety, depression, and hopelessness. Based on the existing research, we propose the following hypotheses:

H1: Higher levels of loneliness (isolation, relational connectedness, and collective connectedness) will be positively associated with greater levels of anxiety, depression, and hopelessness.

H2: Ego-resilience will be negatively associated with anxiety, depression, and hopelessness.

H3: Ego-resilience will mediate/moderate the relationship between loneliness (isolation, relational connectedness, and collective connectedness) and internalizing disorders (anxiety, depression, and hopelessness).

## 2 Materials and methods

### 2.1 Participants and procedures

Participants were young adults (*n* = 337; mean age = 21.95, *SD* = 4.68) enrolled at a metropolitan university in Cape Town, South Africa. The majority of the sample were women (77.2%) and resided in an urban area (75.8%).

We used Google Forms to develop an electronic version of the measures described in the Instruments section. Access to personal information is strictly regulated in South Africa through the Protection of Personal Information Act; therefore, we contacted students via the office of the Registrar of the University. The Registrar emailed a random sample of 1, 200 students a description of the study and an invitation to participate, as well as the electronic link. The survey was conducted through the university’s internal network, which requires authenticated access using university-provided credentials. This meant that only registered students with valid credentials could participate in the survey. The response rate to the invitation to participate was approximately 22%.

### 2.2 Instruments

Participants completed a brief demographic questionnaire, as well as the following standardized questionnaires: the UCLA-LS, the Ego-Resilience Scale [ER89: (37)], the trait scale of the State-Trait Anxiety Inventory [STAI-T: (38)], the Center for Epidemiological Studies Depression Scale [CESD: (39)], and the Beck Hopelessness Inventory [BHI: (14)].

The UCLA-LS is a 20-item measure of an individual’s general sense of loneliness and social connectedness. It provides a score for overall sense of loneliness and three individual components of loneliness. In the current study, only the scores of the components of loneliness were used to determine whether they are differentially related to internalizing disorders. The UCLA-LS items are scored on a 4-point Likert scale ranging from “*I never feel this way*” (1) to “*I always feel this way*” (4). Higher scores on the three components reflect a greater feeling of being alone and disconnected from others (isolation), less satisfaction with close personal friendships (relational connectedness), and less fulfillment in feeling part of a significant group (collective connectedness). The authors of the scale reported a satisfactory reliability coefficient (*α* = 0.94) and provided evidence for concurrent and discriminant validity (2). South African studies have reported satisfactory reliability coefficients (*α* > 0.80) in both a student (40) and teacher sample (41).

The ER89 is a 14-item measure of individuals’ ability to adapt behavior to different situational contexts; thus, it represents adaptation in the face of trauma and stress. The 14 items are scored on a 4-point scale ranging from “*does not apply*” (1) to “*applies very strongly*” (4). Higher scores on the ER89 represent better adaptation in the face of stress. The authors of the scale reported an internal consistency estimate of 0.76 at two different time points in a longitudinal study (37). A study in Africa reported a modest reliability (*α* = 0.64) in a sample of Kenyan children (42). A more recent study of Chinese college students reported a reliability coefficient of 0.72 and provided evidence of criterion-related validity (43).

The STAI-T is a 20-item measure of trait anxiety scored on a 4-point scale ranging from “*almost never*” (1) to “*almost always*” (4). Higher scores on the STAI-T reflect greater levels of anxiety. In the STAI-T manual, the authors reported internal consistency estimates that ranged from 0.86 to 0.92 (38). A reliability generalization study found an average internal consistency estimate of 0.89 (*SD* = 0.05) across 51 studies that used the STAI-T (44). In South Africa, satisfactory reliability coefficients have been reported for the STAI-T when used in a sample of teachers [α and ω = 0.91: (45)].

The CESD is a 20-item measure of depressive symptoms, such as restless sleep, feelings of sadness, and poor appetite. The 20 items are scored on a 4-point scale ranging from “*rarely or none of the time*” (0) to “*most or all of the time*” (3). High scores on the CESD reflect greater depressive symptoms. The author of the scale reported estimates of internal consistency (*α*) ranging from 0.85 to 0.90 and provided evidence of construct validity (39). In South Africa, the CESD has demonstrated satisfactory reliability in a student [α = 0.90: (46)] and teacher sample [α = 0.92, ω = 0.93: (47)].

The BHI is a 20-item measure of hopelessness and pessimistic future expectations. It is scored on a dichotomous measure of “*true*” (1) or “*false*” (0). Higher scores on the BHI reflect higher levels of hopelessness and pessimism. The author of the BHI reported an internal consistency coefficient of 0.93 for the scale and provided evidence of construct validity (14). In South Africa, satisfactory reliability coefficients have been reported in a student sample [α = 0.82: (48)] and a teacher sample [α and ω = 0.89: (49)].

### 2.3 Ethics

The study was approved by the Humanities and Social Sciences Research Ethics Committee of the University of the Western Cape (Ethics reference number HS20/5/1 1 June 2020) and conducted according to the guidelines of the Declaration of Helsinki. Participation in the study was voluntary, and participants provided informed consent on the landing page of the electronic link. No personal data was collected, and no incentives were offered for participation.

### 2.4 Data analyses

All statistical analyses were conducted using IBM SPSS for Windows, version 28 (IBM Corp., Armonk, NY, USA). There were no missing data because the responses to all items of the electronic questionnaire were marked as compulsory, and participants could only proceed if they had responded to all the items on a particular screen. We first determined indices of skewness and excess kurtosis to examine whether the data were normally distributed. Data would be considered approximately normally distributed if skewness values range from −2 to +2 (50) and excess kurtosis values range from −1 to +1 (51). We further obtained descriptive statistics (means and standard deviations) and reliabilities (*α* and *ω*) for all study variables, as well as the intercorrelations (Pearson’s *r*) between variables.

Since a variable can be both a moderator and a mediator (52), mediation and moderation analyses were conducted using the PROCESS macro developed by Andrew Hayes for SPSS (53). In the mediation model, the components of loneliness were used as the predictor variables, ego-resilience as the mediator, and internalizing disorders as the dependent variables. The significance of the indirect effect of the components of loneliness on internalizing disorders through ego-resilience was examined using 95% bootstrapped confidence intervals.

In the moderation analysis, the variables used to create an interaction term (components of loneliness X ego-resilience) were mean-centered to mitigate potential multicollinearity between the predictor and the interaction term. A significant interaction term is indicative of a moderating effect, and the significance of the interaction term was evaluated using 95% bootstrapped confidence intervals. To determine the nature of any significant interaction, we plotted the slopes of the regression line at three different values of ego-resilience: 1 SD below the mean (low level of ego-resilience); the mean (medium level of ego-resilience); and 1 SD above the mean (high level of ego-resilience). The three values chosen provide information about the relationship between the components of loneliness and internalizing disorders only at those particular scores. We supplemented this information by using the Johnson-Neyman technique (54) to determine the exact ego-resilience score at which the relationship between the predictor and dependent variable ceases to be significant. To implement the Johnson-Neyman method, we used a freely available Excel workbook, CAHOST (55), to plot the slope of the regression line for different values of ego-resilience.

## 3 Results

The intercorrelations between study variables, indices of skewness and kurtosis, descriptive statistics, and reliabilities are reported in **Table 1**.

**Table 1:** Intercorrelations between study variables, descriptive statistics, and reliabilities.

Variable	1	2	3	4	5	6	7
1. Collective connectedness	—						
2. Isolation	.45^**^	—					
3. Relational connectedness	.67^**^	.54^**^	—				
4. Ego-resilience	−.51^**^	−.29^**^	−.42^**^	—			
5. Anxiety	.51^**^	.62^**^	.56^**^	−.57^**^	—		
6. Depression	.43^**^	.53^**^	.45^**^	−.42^**^	.79^**^	—	
7. Hopelessness	.48^**^	.43^**^	.53^**^	−.48^**^	.62^**^	.56^**^	—
Mean	8.61	29.85	10.64	41.36	48.12	27.50	4.72
*SD*	2.70	7.38	3.65	6.75	10.53	13.36	4.43
Skewness	0.36	−0.29	0.39	−0.33	0.25	0.02	1.24
Kurtosis	−0.32	−0.60	−0.48	−0.42	−0.17	−0.95	0.95
Alpha	0.78	0.91	0.86	0.82	0.90	0.92	0.88
Omega	0.79	0.91	0.87	0.83	0.91	0.92	0.88

^**^ *p* < 0.001.

**Table 1** reflects that the skewness values ranged from −0.33 to 1.24, while the kurtosis values ranged from − 0.95 to 0.95, which indicates that the data are approximately normally distributed. The estimates of internal consistency for all the scales was satisfactory (*α*: 0.78–0.92; *ω*: 0.79–0.92). The findings in **Table 1** demonstrate that the three components of loneliness were positively related to the three internalizing disorders and negatively related to ego-resilience. The correlations were largely moderate (0.30–0.49), except for the relationships between collective connectedness and anxiety (*r* = 0.51, *p* < 0.001), isolation and depression (*r* = 0.53, *p* < 0.001), and relational connectedness and hopelessness (*r* = 0.53, *p* < 0.001), each of which represented a substantial association (*r* >.50).

The mediation analysis results regarding the direct and indirect effects of the three components of loneliness on internalizing disorders are reported in **Table 2**.

**Table 2:** Direct and indirect effects of the components of loneliness on internalizing disorders.

Effect	*B*	*SE*	95% CI	*β*	*p*
Direct effects					
Collective connectedness → anxiety	1.17	0.20	[0.79, 1.55]	0.30	<0.001
Collective connectedness → depression	1.45	0.27	[.0.92, 1.99]	0.29	<0.001
Collective connectedness → hopelessness	0.53	0.09	[0.36, 0.70]	0.32	<0.001
Isolation → anxiety	0.72	0.06	[0.61, 0.83]	0.50	<0.001
Isolation → depression	0.81	0.08	[0.64, 0.97]	0.45	<0.001
Isolation → hopelessness	0.19	0.03	[0.13, 0.25]	0.32	<0.001
Relational connectedness → anxiety	1.13	0.13	[0.87, 1.39]	0.39	<0.001
Relational connectedness → depression	1.21	0.19	[0.84, 1.58]	0.33	<0.001
Relational connectedness → hopelessness	0.49	0.06	[0.37, 0.60]	0.40	<0.001
Indirect effects					
Collective connectedness → ego-resilience → anxiety	0.80	0.13	[0.57, 1.06]	0.21	—
Collective connectedness → ego-resilience → depression	0.69	0.15	[0.41, 0.99]	0.14	—
Collective connectedness → ego-resilience → hopelessness	0.26	0.05	[0.17, 0.37]	0.16	—
Isolation → ego-resilience → anxiety	0.17	0.04	[0.10, 0.25]	0.12	—
Isolation → ego-resilience → depression	0.16	0.04	[0.09, 0.24]	0.09	—
Isolation → ego-resilience → hopelessness	0.07	0.02	[0.04, 0.10]	0.11	—
Relational connectedness → ego-resilience → anxiety	0.48	0.08	[0.33, 0.65]	0.17	—
Relational connectedness → ego-resilience → depression	0.44	0.10	[0.27, 0.64]	0.12	—
Relational connectedness → ego-resilience → hopelessness	0.16	0.03	[0.10, 0.23]	0.13	—

**Figure 1:**
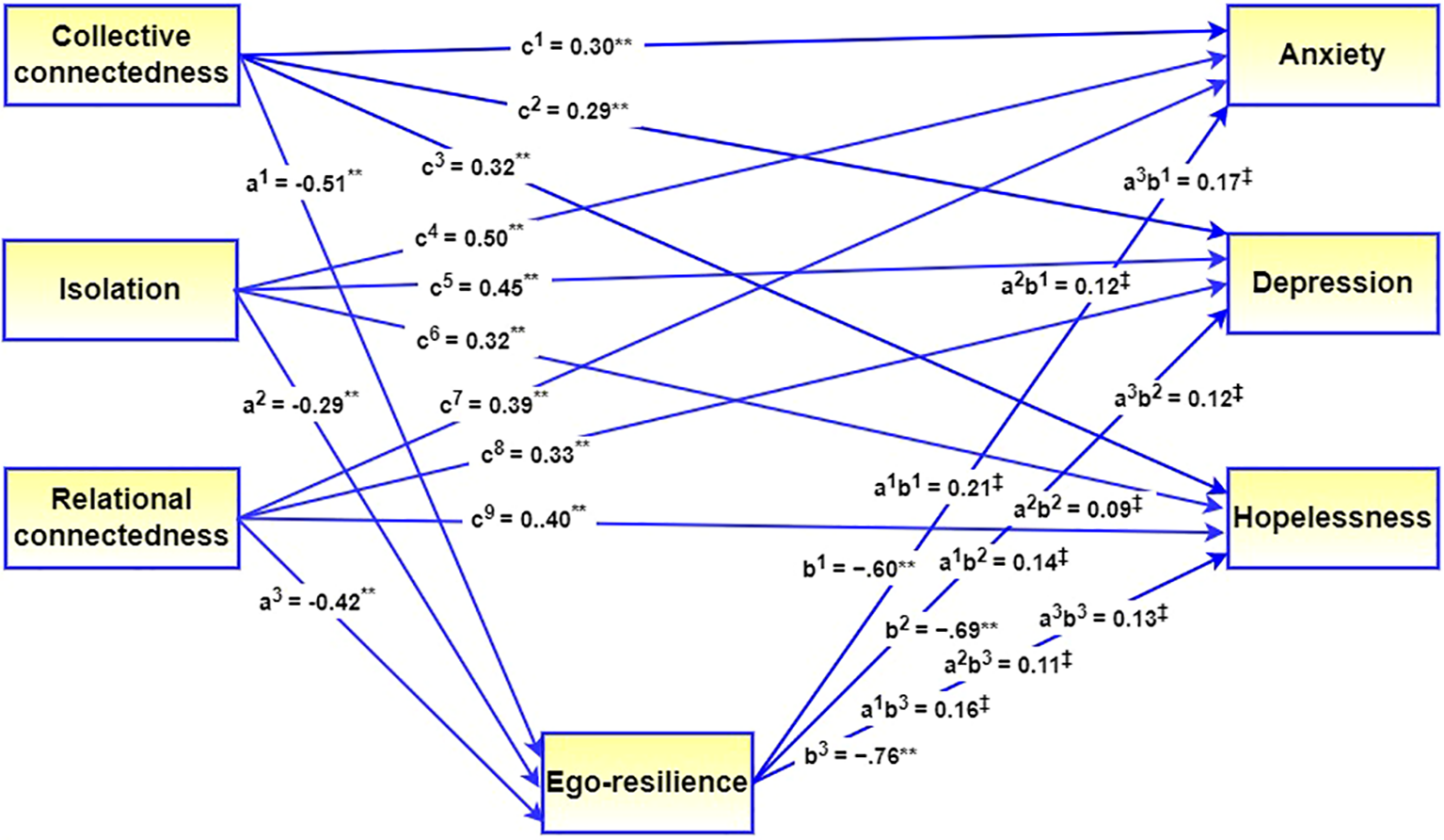
Visual representation of the mediating role of ego-resilience in the relationship between the components of loneliness and internalizing disorders. c^1^–c^9^ = direct effects of predictor on dependent variables, a^1^–a^3^ = direct effect of predictor on mediator, b^1^–b^3^ = direct effects of mediator on dependent variables, a^1^b^1^– a^3^b^3^ mediating effects. All regression coefficients are standardized. **p < 0.001, ^‡^95% CI.

Regarding direct effects, the *p*-values in **Table 2** indicate significant effects of the three components of loneliness on anxiety, depression, and hopelessness. Further, the findings in **Table 2** indicate that ego-resilience was the pathway through which the three components of loneliness impacted anxiety, depression, and hopelessness, because the 95% bootstrapped confidence intervals for all indirect effects did not contain zero. In the absence of the mediator, a significant association was observed between collective connectedness, on the one hand, and anxiety (*β* = 0.51, *p* < 0.001), depression (*β* = 0.43, *p* < 0.001), and hopelessness (*β* = 0.48, *p* < 0.001), on the other hand. Similarly, isolation was significantly associated with anxiety (*β* = 0.62, *p* < 0.001), depression (*β* = 0.53, *p* < 0.001), and hopelessness (*β* = 42, *p* < 0.001), and relational connectedness was significantly associated with anxiety (*β* = 0.56, *p* < 0.001), depression (*β* = 0.45, *p* < 0.001), and hopelessness (*β* = 0.53, *p* < 0.001). In the presence of the mediator, these associations remained significant but all coefficients were reduced. This finding is indicative of partial mediation. The mediation model is visually presented in **Figure 1**.

The significance of the interaction terms in the moderation analyses are presented in **Table 3**.

**Table 3:** Significance of the interaction terms in the moderation analyses.

Interaction term	*B*	*SE*	95% CI	*p*
Collective connectedness X ego-resilience → anxiety	0.02	0.02	[−0.02, 0.07]	0.35
Collective connectedness X ego-resilience → depression	0.00	0.03	[−0.06, 0.07]	0.95
Collective connectedness X ego-resilience → hopelessness	−0.02	0.10	[−0.04, −0.01]	0.02^*^
Isolation X ego-resilience → anxiety	−0.00	0.01	[−0.02, 0.01]	0.66
Isolation X ego-resilience → depression	0.01	0.01	[−0.01, 0.03]	0.49
Isolation X ego-resilience → hopelessness	−0.01	0.00	[−0.01, 0.00]	0.14
Relational connectedness X ego-resilience → anxiety	−0.00	0.02	[−0.04, 0.03]	0.93
Relational connectedness X ego-resilience → depression	−0.02	0.03	[−0.07, 0.03]	0.37
Relational connectedness X ego-resilience → hopelessness	−0.02	0.01	[−0.03, −0.00]	0.02^*^

^*^ *p* < 0.05.

**Figure 2:**
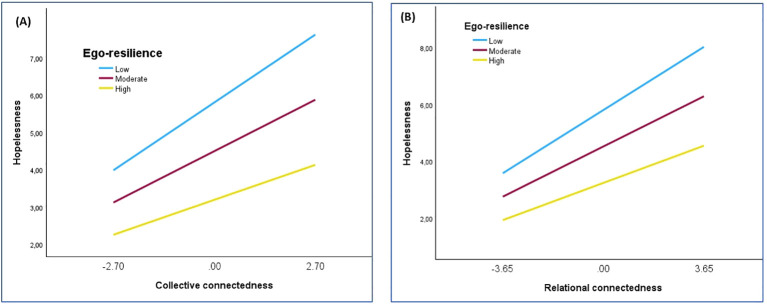
Relationship between collective connectedness **(A)**, as well as relational connectedness **(B)**, and hopelessness for low, moderate, and high levels of ego-resilience.

The findings in **Table 3** demonstrate that collective connectedness and relational connectedness interacted with ego-resilience to impact hopelessness. All other interaction terms did not significantly impact the internalizing disorders. The nature of the two moderating effects is demonstrated in **Figure 2**, which plots the relationship between collective connectedness and relational connectedness, respectively, and hopelessness for three different ego-resilience scores: 1 *SD* below the mean, the mean, and 1 *SD* above the mean.

**Figure 3:**
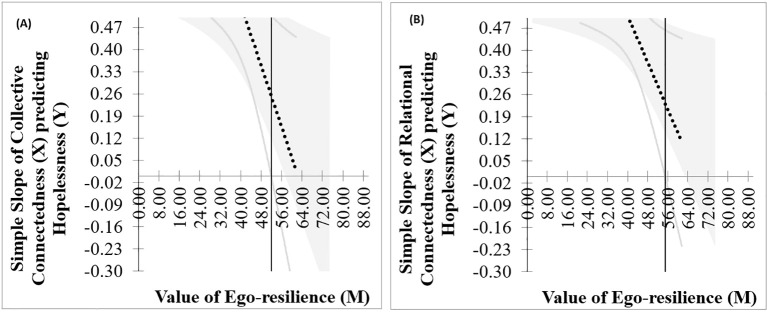
Johnson-Neyman plot of the relationship between collective connectedness **(A)**, as well as relational connectedness **(B)**, and hopelessness for a range of ego-resilience values.

**Figure 2** demonstrates that the relationship between collective connectedness (A) and relational connectedness (B) was stronger at lower levels of ego-resilience than at higher levels of ego-resilience. At both high and low levels of collective connectedness, as well as high and low levels of relational connectedness, a higher level of ego-resilience was associated with a lower level of hopelessness. The Johnson-Neyman plot in **Figure 3** demonstrates the change in the relationship between collective connectedness, as well as relational connectedness, and hopelessness over a range of ego-resilience values.

**Figure 3** demonstrates that the slope of the regression line for the relationship between collective connectedness, as well as relational connectedness, and hopelessness decreases as the value of ego-resilience increases. In the Johnson-Neyman plot, the gray area represents the confidence interval around these slopes, and the vertical line represents the value of ego-resilience where the confidence intervals contain zero, which indicates that the relationship between the predictor and dependent variable is no longer significant. The value for the relationship between collective connectedness and hopelessness was 51.94, and the value for the relationship between relational connectedness and hopelessness was 54.98. Above these values, the relationship between the predictors and dependent variable would not be considered significant.

## 4 Discussion

This study extends previous research by exploring how various aspects of loneliness—isolation, relational connectedness, and collective connectedness—interact with ego-resilience to influence anxiety, depression, and hopelessness. There were several important findings. First, the three components of loneliness were positively related to the three internalizing disorders and negatively related to ego-resilience. Specifically, collective connectedness showed a significant relationship with anxiety, isolation was closely linked to depression, and relational connectedness was strongly associated with hopelessness.

Connectedness with the social world reflects an internal sense of belonging and a subjective awareness of being in close relationship with other people. It is the aggregate of social experiences (e.g., with family, friends, peers, school, and community) that is internalized by the individual over time and forms the basis for the experience of connection and relation with others. This enduring sense of connectedness can be conceptualized as a relational schema or framework that guides cognitions, feelings, and behaviors, particularly in the context of interpersonal relationships. People with low levels of connectedness are likely to view the social world as threatening, mistrust interpersonal relationships, and avoid or retreat from social interactions. A worldview that is shaped by low connectedness can precipitate a cycle of negative interactions and perceptions that further entrench disconnection and alienation. This negative cycle can impact an individual’s self-concept and their ability to use the social environment for emotional coping, which may contribute to adverse mental health outcomes (56, 57). Evidence for this phenomenon drawn from a range of studies (58–60) may explain the association between the three components of loneliness and anxiety, depression, and hopelessness, respectively.

Second, the mediation analysis demonstrated that ego-resilience is the pathway through which loneliness affects anxiety, depression, and hopelessness. Ego-resilience also moderated the impacts of collective connectedness and relational connectedness on hopelessness. This finding corresponds with the findings of previous studies highlighting the role of ego-resilience as a protective resource. Philippe and colleagues (61) reported that ego-resilience mediated the relationship between exposure to trauma in childhood and the development of anxiety, depression, and self-harming behaviors. Alessandri and colleagues (62) found that ego-resilience mediated the longitudinal relationship between socioeconomic status and school grades among children. In a study of patients with rheumatoid arthritis, Ziarko and colleagues (63) reported that ego-resilience mediated the relationship between emotion-oriented coping and life satisfaction. Similarly, Kim and colleagues (64) observed that ego-resilience was a partial mediator of the relationship between acculturation stress and mental health among North Korean refugee youth.

Third, the results suggest a critical threshold value for ego-resilience, beyond which the relationship between components of loneliness and hopelessness becomes statistically nonsignificant. The identified threshold values suggest that there is a level of ego-resilience that may sufficiently buffer an individual against the impact of loneliness, rendering its interaction with hopelessness insignificant. For example, when ego-resilience scores surpass these thresholds, the strength of the predictive relationship between collective connectedness, relational connectedness, and hopelessness decreases to a point of nonsignificance. Understanding these threshold values may enable clinicians and mental health practitioners to design and implement more precise goals for resilience training programs, aiming to elevate individuals’ resilience to or beyond the identified threshold. Various studies have supported the efficacy of cognitive behavioral therapy in enhancing ego-resilience and promoting adaptive coping (64, 65). By incorporating strategies focused on building ego-resilience, such interventions can effectively mitigate the psychological impacts of loneliness on internalizing disorders. Resilience-building workshops in community and organizational settings could aim to raise individuals’ resilience levels beyond the critical threshold identified in this study, effectively buffering against the adverse effects of loneliness on hopelessness. Such interventions would promote mental health and well-being and provide individuals with the tools to maintain long-term psychological resilience in the face of stressors.

The study has certain limitations. The cross-sectional design limits inferences related to causality, limiting conclusions about the directionality of the relationships between loneliness, ego-resilience, and internalizing disorders. The sample consisted of young adults studying at a university in South Africa and the factors that influence experiences of loneliness and the development of ego-resilience in this population may differ substantially from those in other regions or among individuals who are not in higher education. For instance, the South African context is shaped by a unique history of social fragmentation due to apartheid-era policies, as well as current challenges such as high unemployment, violent crime and widespread poverty. These factors may heighten experiences of isolation or collective disconnection, influencing how loneliness and ego-resilience interact to affect mental health outcomes. Furthermore, cultural norms around individualism versus collectivism could significantly shape the experience of loneliness and its relationship with internalizing disorders. In collectivist cultures, relational and collective connectedness may have a more salient role in buffering against loneliness compared to more individualistic societies. Cultural factors have also been found to have a significant role in cognitive appraisals of experiences and the resultant development of mental health disorders (66). These cultural differences highlight the need for caution in generalizing the findings of this study to other populations. Another limitation of the study is the use of self-report measures, which are susceptible to social desirability and respondent bias. The use of moderation in a cross-sectional study can be a limitation due to the inability to establish temporal precedence. In the absence of longitudinal data, it is challenging to determine whether the moderator influences the strength or direction of the relationship or whether the observed interaction is the result of other confounding factors. Future studies of a longitudinal design are recommended to corroborate the findings of the study. The low response rate (22%) represents a notable limitation of this study. It can introduce potential biases, particularly non-response bias, which may affect the generalizability of the findings. It is recommended that future studies consider implementing strategies to improve response rates, such as offering incentives for participation. In addition, using data triangulation techniques could help to validate and contextualize findings obtained from low-response survey.

Despite these limitations, the present study provides compelling evidence for the protective role of ego-resilience in specific internalizing disorders.

## 5 Conclusion

The study expands the existing knowledge base on the role of components of loneliness in specific internalizing disorders and demonstrates the influence of ego-resilience on these relationships. The results suggest a nuanced view of loneliness as a significant factor capable of shaping mental health trajectories through interactions with individual personality traits like ego-resilience. Understanding the mediating and moderating role of ego-resilience can inform therapeutic approaches and interventions aimed at reducing the impact of loneliness on mental health.

## Data Availability

The raw data supporting the conclusions of this article will be made available by the authors, without undue reservation.
